# Patients’ perception of medical care in the hospital environment: the reasons of non-hospitality

**DOI:** 10.1186/s13010-025-00176-0

**Published:** 2025-07-16

**Authors:** Laura Marques Castelhano, Gilberto de Araujo Guimarães, Isabel Baptista

**Affiliations:** Faculty of Education and Psychology, Research Centre for Human Development, Universidade Católica Portuguesa, Portugal Campus Porto, Rua de Diogo Botelho, 1327, Porto, 4169-005 Portugal

**Keywords:** Healthcare hospitality, Hostility, Physician-patient relationship, Patient perception, Physician attitudes

## Abstract

**Background:**

The medical care provided by the physician is an important part of the hospital scene and the action of caring. Assessments of the physician-patient meeting are based on welcome and the physician’s ability to be perceived as hospitable by the patient. By definition, to be hospitable is to have the ability to welcome, care for, reassure, and be courteous, respectful, and trustworthy. This article aims to understand patients’ perceptions of medical care perceived as not hospitable, characterized by a lack of care and welcome, in a hospital environment, based on a complaint’s website.

**Method:**

The research method used was qualitative analysis and the research strategy was documentary research. The data were collected on a complaints registration platform. The theoretical framework used was the theory of Hospitality. The study selected, coded, and categorized the complaints of 127 patients at the 09 most renowned private hospitals in Brazil. The Voyant tools assisted in the textual analysis of complaints while coding classified them into categories.

**Results:**

After evaluating the reasons and elements of the complaint, the following was analyzed the encounter characterized as hostile and inhospitable and the attitudes perceived by the patients were grouped into what was defined as “the 4 D’s of non-hospitality”: dehumanization, disregard, dereliction of duty, and disability. Each of the attitudes was characterized by the physician’s behavior and the sensations, emotions, and feelings triggered in the patient.

**Conclusions:**

Patients’ perception of the not hospitable encounter may be hostile or inhospitable. The physician’s attitude is an important criterion for evaluating the encounter. The physician’s attitude and the form of care are key factors in a culture focused on hospitality in the hospital environment. Hostile and inhospitable attitudes affect the physician-patient relationship and may compromise the patient’s well-being.

## Introduction

What is the patient’s perception of medical care that is considered not hospitable?

When a patient enters a hospital they bring expectations of supportive treatment and care. They yearn for acceptance as fear and uncertainty are often present. The physicians plays a fundamental role in care and must, as part of their practice, ensure that the hospital is a safe and welcoming place. The hospital is a place of fragility. Fragility of patients, healthcare professionals and the healthcare system. Schaad [1, p. 13] asks “What if hospitality were not an anecdotal issue, but a fundamental element of the future of the healthcare system?”. Changes can be born from tensions and paradoxes.

Hospitals in Brazil, and around the world, have expanded their method and capacity of care by creating more spaces for comprehensive patient care, providing not only emergency room care, and hospitalization for treatment and surgery, but also consultations and exams, thus recovering the original concept of a place of welcome, treatment and healing.

Studies on healthcare hospitality focus on reading the spaces, services, and environment, but do little to analyze the way physicians act, and their relationship with patients, as an important part in the process [2–7].

Some approaches are made in the works by Boeger [8] and Godoi [9], which reflect on the potential influence of physicians in healthcare hospitality. Yang and Kirillova [10] discuss the importance of creating a ‘culture of hospitality’ in healthcare, where the physician is responsible for providing and maintaining professional integrity, guaranteeing the patients “hospitable care” in a safe environment. According to Poorani, Kline, DeMicco and Sullivan [11], an understanding of hospitality enhances the physician’s motivation to build emotional relationships by incorporating the human experience into the healthcare environment. Other works such as Perezim and Camargo [12], who discuss the relationship between hospitality and death, Meneses [13], who discusses the issue of humanization in health and hospitality from a philosophical point of view, Reach [14], who addresses patients’ expectations regarding quality and safety, and Hunter-Jones, Sudbury-Riley, Al-Abdin and Spence [15], who points out how hospitality can play an important role in palliative care.

According to Benveniste [16] *hospitality*,* hospitalitate* in Latin, meant the act of receiving, hosting, and, by extension, the safe refuge. *Hospitalitate*, in medieval Latin, became *hospitale*, which gave rise to the term hospital *(hôpital*), a place of refuge and treatment in modern Latin languages. *Hostis* meant equality by compensation, the one who compensates for a gift with a counter-gift. However, it also meant hostile, the opposite, the adverse, the enemy. Hostility seems to be the opposite of hospitality, in that to be hostile is to treat someone as an enemy, to cause harm, to damage [17].

The origin of expressions that refer to doctors, medics or physicians comes from the Latin words “mederi”, “physica” or “doctor”, which mean “knowing the best way” for something, reflecting the idea that a physician knows the way for the cure [16].

The action of the physician, an important actor in the hospital health system, by providing a space for welcome and care for the patient, perpetuates a culture of hospitality. However, similarly, an imbalance in the relationship of the physician and patient, can perpetuate a hostile culture with consequences for the system, institutions and patient health.

According to Fleury [18], no patient when seeking hospital care wants to choose between empathy and competence of the physician. Physicians are called to provide what is essential for the treatment and establishment of a relationship of trust.

The problem of the physician-patient relationship should orientat actions, research and proposals for improvements in various areas, including the discussion about the training of the physician.

In their professional work, when meeting patients, physicians need to consider the relational aspects and the responsibilities and implications of this interaction. Evaluations of the meeting are established in the relationship by the physician’s welcome and the ability to be perceived as hospitable by the patient.

This article aims to understand patients’ perceptions of medical care perceived as not hospitable, characterized by a lack of care and welcome, in a hospital environment, based on a complaint’s website.

## Theoretical background

### Hospitality and hostility

The concept of hospitality, care, and welcome, can broaden discussions about the physician’s role in patient care and the consolidation of a health system.

According to Baptista [19], hospitality is a privileged mode of interpersonal meeting, defined by a welcoming attitude towards others. To Guimarães [20], hospitality is a relationship that takes the form of an interpersonal meeting marked by acceptance and welcome and which produces connection. Connections are important for building bonds of trust that can trigger feelings of support and care, producing adherence and autonomy in treatment. Welcoming is hope, therefore hospitality and humanization are about waiting and hoping [13].

According to Lashley [21] hospitality is about relationships and therefore intersubjectivity. Intersubjectivity is the connection between people, making them sensitive to each other’s emotional world.

The hospitable meeting in the physician’s care is the basis from which relationships are created, as it allows the patient to establish or extend the bond with the physician in a concrete way. According to Camargo [22], defining the different forms of human relationships as the purpose of studying hospitality and evaluating the result from the point of view of strengthening or weakening the bond shows that hospitality concerns all relationships between human beings.

For Kant [23], in a broader view, hospitality can be seen as a moral duty. You have to exercise the duties of virtue with others. Treat others well as a duty, not out of pity or compassion. A duty that is owed by the host concerning the supreme principle of morality [23].

Levinas [24] goes beyond duty and right. For him, hospitality is an ethical-theological issue, an ethical responsibility, eminently personal, integrated into an ethics of the face or ethics of responsibility. It is about a self that is responsible for the other. The ethics of Levinas [24]. unlike Kantian ethics, is a kind of original awakening of a “self” responsible for the “other”.

For hospitality to be considered and evaluated as an intrinsic characteristic, it would have to be established beyond social impositions. This is how Derrida proposes genuine, unconditional hospitality, in an antinomy between the unconditionality of total openness to the other who arrives and the rules and laws of hospitality, conditional rights and duties, unconditional hospitality would be above the laws [25].

For Kant [23], man is a social being, but he is also antisocial. From his perspective, duality is characteristic of human nature. As a gregarious animal, he tends to relate to others, but he also tends to isolate and withdraw. From this process of attraction and repulsion, which is typical of human sociability, the notion of antagonism and hostility emerges as a cause of wars but also of progress. The development of all dispositions is carried out by Nature through their antagonism in society, which ultimately leads to the establishment of a legal order for these dispositions.

According to Camargo [22, p. 47], hospitality intersects four concepts: human relationship, virtue, ritual, and exchange and they must be understood as a “human relationship in which an exchange occurs between the host and the guest, which may result in appeasement, positive feelings [.] friendship, love, human warmth [.] and even a certain level of conflict, aggressiveness, and hostility”.

The author considers attitudes that do not align with hospitality as inhospitable when there is no interest in contact with others, as well as a failure to give and receive. and, in many cases, this can even lead to hostility, resulting in mistreatment and hostile feelings. It is as if there was a *continuum* of attitudes between hospitality and hostility, with inhospitality being the dividing line between them both.

*Designating hospitality as a virtue is to consider that the social panorama is marked by its absence. Thus*,* in the same way that hospitality is considered a virtue and is often associated with terms such as sociability*,* solidarity*,* charity*,* love*,* etc. its absence is marked by terms such as inhospitality*,* misanthropy*,* hostility*,* aggression*,* violence*,* parasitism*,* ostentation*, etc [22, p. 51].

In turn, according to Alkan [26], hostility, which is also seen as an attribute of relationships, occurs simultaneously with flows of generosity and solidarity, but hostility is not an immediate quality of hospitality. These two ways of relating to others can be closely related, but their relationship is not deterministic. For Alkan [26] hospitality is not simply opening the door to the Other; it is also opening oneself to the perspective and dangers of a relationship. This relationship can turn into anything a relationship can turn into. Their initial asymmetries can survive the relationship and lead to hostilities of various kinds, but they can also lead to intimate relationships.

According to Santos, Perazzolo and Ferreira [27] hostile people essentially talk about refusing to welcome and feeling threatened in their support system; they talk about having received nothing (giving) or exchanged nothing (receiving and giving back) and feeling invaded; they complain about the imposition of presences and customs, instead of taking the new as a unique opportunity to learn, they complain about the lack of consideration for those who already inhabited the place, the lack of negotiations and explanations. Hostile people refer to the discomfort that arises from the feeling of being robbed of the space they believe they have and belong to.

As emphasized by Guimarães e Camargo [28] the welcome and acceptance is the result of a choice between accepting or rejecting the other. The other will be accepted if perceived as similar and not threatening. It will be rejected if perceived as strange and threatening.

Hospitality is the right not to be treated as an enemy when arriving in another’s land. The other is the stranger to everything that is not “me” [29].

### Hospitality and its importance in the physician-patient relationship

Relationships are marked by perceptions and the experience of meeting others. An experience that must be “taken care of” from the physician’s point of view. In a perspective that broadens the notion of “care”, in a binding framework, the physician must be concerned with welcoming. Welcoming complaints, welcoming doubts, welcoming emotional demands, and welcoming the other person in their essence.

Botschen [30, 31] proposes a classification of meetings to provide services based on the intensity of the bonds established. For him, the first type of meeting is the direct personal one, in which there is a high degree of human interaction and a strong intensity of bonds. This is the type that characterizes the meeting between physician and patient. King [31. p. 229] analyzing the hospitable meeting, says that “values such as fraternity and friendship, respect and reverence, tact and polish are fundamental to this task”.

Majeed and Kim [3] see the physician taking on an important role in personalized and attentive patient care, ensuring a sense of psychological comfort, safety, and polite communication, which, together with technical qualifications, creates an experience that is perceived as positive and results in the patient’s rapid recovery. According to Poorani, Kline, DeMicco and Sullivan [11] successful medical treatment, wrapped in the empathy, cheerfulness, courtesy, responsive attitude, and quality communication of a health professional, is an attractive attribute of the healthcare service to boost the patient experience.

For Godovykh and Pizam [32], Drossman, Palsson, Stein, Ruddy and Lennon [33] and Costa, Moura, Moraes, Santos and Magalhães [34], understanding the patient experience is crucial, as it influences patient satisfaction, the perceived quality of health services, loyalty to physicians, and service providers, as well as patient health and well-being. For the authors, the quality of the physician-patient relationship is the main driver of patient satisfaction with his care.

Multiple variables can affect the physician-patient relationship, but its intensity and importance are anchored, from the physician’s point of view, in two principles: technical conduct and human understanding.

According to Milford [35], although hospitality is an inviting concept, in contrast it also implies hostility, which suggests the paradox of building a safe place in a strange land and exploits the inherent violence.

Within relational space, hostility produces consequences. Breaks of trust and bond, institutional triggers, propagation of inappropriate behaviors, attitudes and values that culminate in a hostile culture. In addition to the health impact of all actors involved in the system.

A proper intersubjective relationship between physician and patient is fundamental to enabling the treatment of the patient’s health. The issues that may occur in this interaction stem from the difficulty of understanding what the consultation space means.

Campos and Fígaro [36] and Delsart and Auriac-Slusarczyk [37], when researching healthcare and its space for interlocution, showed that it is the physician’s role to negotiate intentions to verify the diagnosis by integrating the patient’s experience.

According to Pazinatto [38], the Federal Medical Council’s recommendation on patient consent and autonomy places the responsibility on the physician to develop an empathetic personal relationship with the patient. In addition, he points out that the precariousness of healthcare in Brazil cannot be justification for a deteriorated physician-patient relationship. According to Minahim [39], the concept of autonomy alone is not enough to serve as the foundation of the physician-patient relationship. Autonomy is a quality attributed to the person, which plays an important role in interpersonal relationships.

The therapeutic alliance, according to Hughes and Kerr [40], is fundamental to treatment in medicine. However, this alliance can be damaged and distorted by the expectations, needs, and desires of both the patient and the physician. Good practice therefore needs to include a questioning attitude and an understanding that physicians and patients are affected in the relationship.

### Patient perceptions and physician’s attitudes

The hospitable or not hospitable encounter involves perceptions, emotions and attitudes. In the physician-patient relationship, these variables are translated by patients into feelings of care or not care offered by the physician.

Perceptions in professional care environment are built according to O’Sullivan and Spangler [41] and Knutson and Beck [42] considering three phases: (1) the pre-meeting; that of creating expectations, by searching for information in all media and previous experiences. These expectations will be the basis for evaluating the service in the post-meeting; (2) the meeting itself, the service provision; (3) the post-meeting; the evaluation made by the perception and evaluation of the delivery, which can be either positive, and therefore generate a new meeting or recommendations to other people, or seen as negative, which will hinder new relationships and generate derogatory comments about the service and the provider.

The evaluation depends not only on how the care was performed, but also on the choices and expectations that the patients had.

Expectation is the establishment of what you want, what you desire. It is the wait for what you want to happen, based on a decision, on choosing a certain alternative. However, it is not limited to just that. According to Ariely [43], the expectations that determine the evaluation of a service can be based on choices made in a “predictably irrational”, more subjective, intuitive, and emotional way, through heuristic processes and biases. Emotional processes can influence attitudes and perceptions and interfere with the physician’s activity [44].

The perception of care with the encounter is also associated with the attitudes of the professional. In principle, perception is divided into three categories: (1) the professional’s attitude towards a problem, or failure to perform the service; (2) the professional’s attitude towards the patient’s needs: (3) the professional’s attitudes that demonstrate a real interest in the patient [45].

When evaluating patient satisfaction, Bernardi [46] pointed out the importance of the perception of the physician’s communicative behavior and concluded that patients feel satisfied when they feel welcomed when they are recognized and called by name, when the physician looks them in the eye and gives them attention, in other words, when they perceive the physician’s interest in their health and gives them information that is appropriate to their level of understanding.

Eigeland, Jones, Sheeran and Moffitt [47], to identify the medical behaviors that patients consider contributing to a good physician-patient relationship, grouped them into six broad domains: (a) valuing the person as a whole, (b) investigation and future planning, (c) collaboration and empowerment, (d) validation and emotional support, (e) politeness and courtesy, and (f) professionalism.

Grundnig, Steiner-Hofbauer, Katz and Holzinger [48], examined the characteristics most considered for evaluation as “good” and “bad” physicians from the patients’ perspective. Social skills and professional competence appeared as the two most important categories in the patients’ perception.

According to Majeed and Kim [3], the patient’s evaluation of the physician depends on a respectful attitude, attentive listening, communication skills, and sufficient time during interactions to address concerns.

For Zygourakis, Rolston, Treadway, Chang and Kliot [49], the importance of hospitality-induced care skills are criteria for a satisfactory patient experience.

Hospitable and not hospitable attitudes can be distinguished, according to Camargo [33, 50] in a *continuum* of hospitality that includes: (a) hostility, which would be the result of aggressive actions that lead to the fraying of human relations; (b) inhospitality, which represents a lack of interest in contact, ignores the other and demonstrates unfriendliness; (c) urbanity, which would represent a pleasant but polished and staged meeting; and (d) hospitality, which would be genuine hospitality that would mark the meeting between people who know and like to receive and be received.

The physician’s behavior and attitudes, especially those considered inadequate, are a risk not only for the physician-patient relationship but as Wang, He, Liu and Li [51]. points out, it is a risk for the entire health system. Bartholomäus, Zomorodbakhsch, Micke et al. [52], when investigating patients’ perceptions of physicians’ ethical behavior, concluded that competence, honesty, respect, and patience are important characteristics that should be improved in medical training.

Yang and Kirillova [10] believe that building a culture of hospitality necessarily involves internalizing significant hospitality practices, defined by physicians in their actions with patients as professionalism, genuineness, transparency, and safety.

For Balint [53], physicians’ attitudes reflect the way they have learned what it means to be a physician. In order to “be a physician”, physicians assume an image that overvalues neutrality and influences distancing attitudes that can result in a lack of understanding of patients’ needs [54].

The physician-patient relationship is the result of a compromise between patients’ requirements and the physician’s attitudes [53]. Physicians need to be aware of their actions and attitudes in the encounter with the patient because these can interfere with the patient’s perception and have consequences in the treatment.

## Method

This study used documentary research as its research strategy, with a qualitative approach, and the theory of Hospitality, as developed by the authors mentioned before, as its interpretation process.

Qualitative approaches, according to Denzin and Lincoln [55], consist of a set of interpretative practices that seek to understand or interpret phenomena in terms of the meanings that people attribute to them.

Documentary research, according to Sá-Silva, Almeida and Guindani [56], is a procedure that uses methods and techniques to collect, understand, and analyze documents that have not received scientific treatment. According to May [57], documentary research produces very valuable insights into societies and the dynamics of social life and allows the researcher to consider not only how meaning is built, but also how new meanings are developed and employed.

The data was collected from a online plaform that registers complaints. Anyone can register a complaint about a service. It is an open and independent platform that acts as a channel between customers and companies. People record their complaints about negative experiences on the institution’s website. Companies have the possibility of getting closer to their customers, understanding their complaints, and directing interventions and possibilities for improvement.

The estudy was carried out on the pages of the 09 most renowned private hospitals in Brazil on the platform. A preliminary analysis selected 1957 complaints that occurred in one year, from 05/01/2022 to 04/30/2023. After the initial analysis, 339 complaints related to the physician’s care were selected. Nonspecific complaints and those unrelated to the healthcare were excluded, leaving 127 complaints to be analyzed.

The health system in Brazil is structured into the public sector, represented by the “SUS”, the Unified Health System, which offers free and comprehensive care to the population, and the private sector, which includes health plans and private services. Many Brazilians turn to the private sector due to the overload and poor service of the SUS.

The corpus composed of 127 complaints was analyzed, considering:

A textual analysis of the corpus and was supported by the software Voyant Tools [58]. Voyant Tools is a text analysis that has tools that support the researcher. The tools used were: Cirrus, the word cloud that shows the most frequent terms; Context, that shows the occurrence of keywords within a context and Topic that generates groupings of terms in related topics. The data collected formed the basis of and complemented the study’s interpretations, along with the analysis of the categories, detailed below.

Next, the data was organized through the analytical coding strategy according to the coding system described by Ezzy [59] containing 4 important stages that occur dynamically.

The first step involved a fluid reading of the data, looking for important points and general characteristics of the problem, and identifying units of analysis: the patient’s perception and medical care not hospitable. The second step aims to name and categorize the phenomenon observed through a detailed examination of the units of analysis. Two conceptual definitions were used as criteria:

- Hostility: the result of aggressive actions that lead to the breakdown of relationships and relational conflicts.
- Inhospitality: an encounter in which the receiver does not recognize and/or ignore the interlocutor; lack of interest in the other.

The third step explored the categories and searched to identify properties and dimensions that characterize them. It is a stage that involves trials and experimentation to identify categories and groupings through comparison and contrasts, looking for similarities and differences in the data regarding the object of study. The fourth stage evalueted the conditions associated with the categories, their properties, and dimensions, identifying codes and connecting them to concepts, themes, and theories.

It should be noted that although the analysis procedure is described sequentially for the sake of clarity and systematization, the analytical process is recursive and dynamic. When interpretations emerge, they can be adjusted and refined.

## Results

The analysis of the 127 complaints, documented on the complaint’s platform, aimed to answer the question: What is the patient’s perception of medical care, in a hospital environment, that is considered not hospitable?

Next, the results will be presented, considering the main findings of the research.

Finding 1: Reasons and elements of the complaint.

We sought to understand what motivated patients’ complaints on the platform, considering this important data to reflect on the patient’s perception of the physician’s care.

**Fig. 1:**
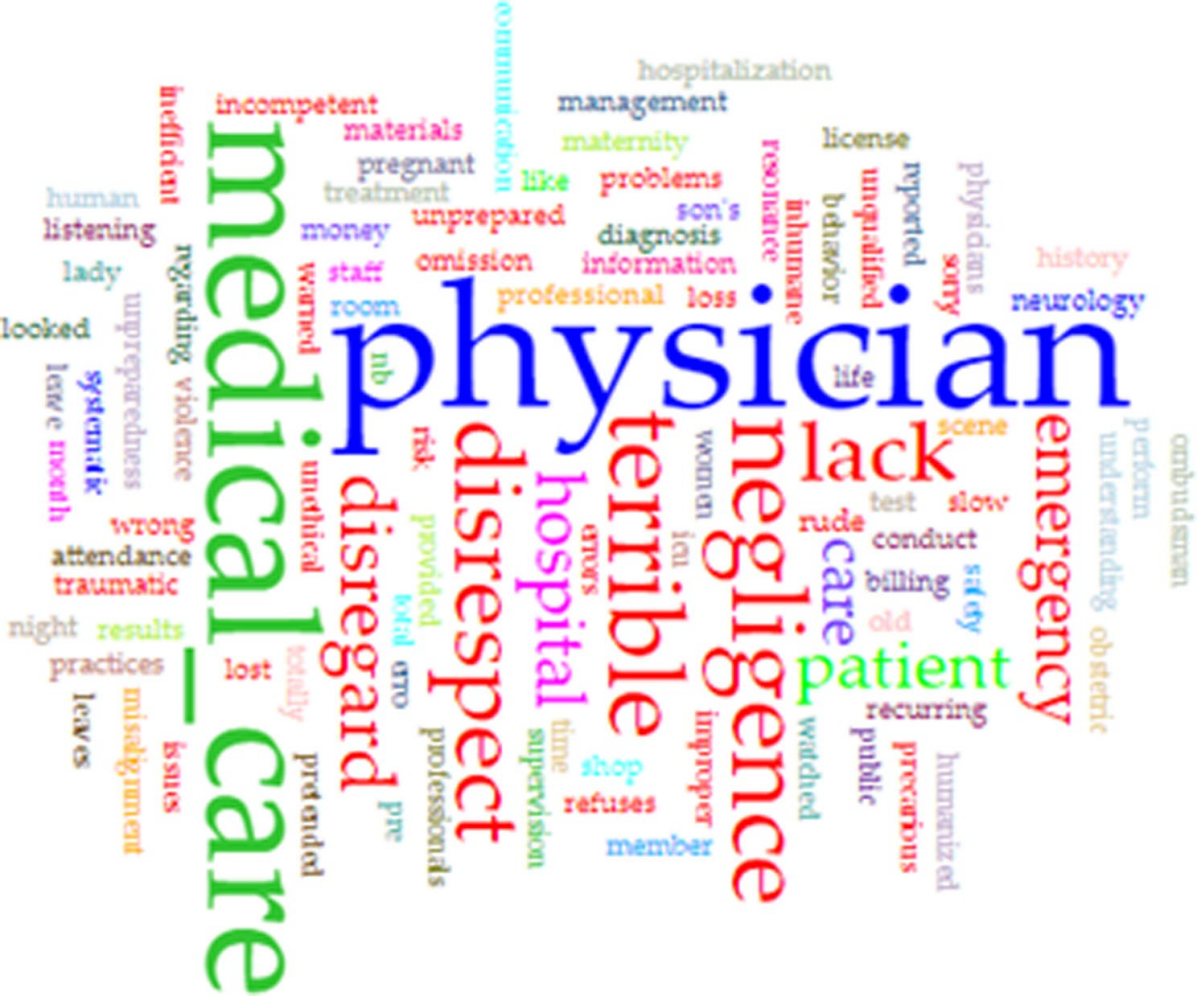
Word cloud. Source: Voyant Tools [58]

The most frequent words identified were: physician, medical care, terrible, negligence, disrespect, disregard, lack, emergency, patient and hospital. (Fig. 1)

Importantly, the fact that “medical care” and “physician” were the most frequent words reinforce the quality of the corpus construction. These two terms and the fact that the words disrespect, disregard and negligence also appear as the most frequent show that the physician’s behavior is an important criterion for patient’s evaluation about the physician’s care.

In the analysis of occurrences related to the keyword, it was chosen to verify in which contexts (frequency and relation) the word physician appears in the corpus. The system groups, by default, 5 words.

It was found that when the word physician appeared in the text, it was related to:

- Perception of the quality of care: terrible, bad service, dehumanized, inefficient.
- Physician’s conduct: negligent, disregard, disrespectful, dangerous, mistake.
- Behavior: bad-mannered, arrogant, not humanized, rude, lack of empathy, doubtful.
- Other aspects: undue billing, delay in service, without clarification, hospitalization.

**Fig. 2:**
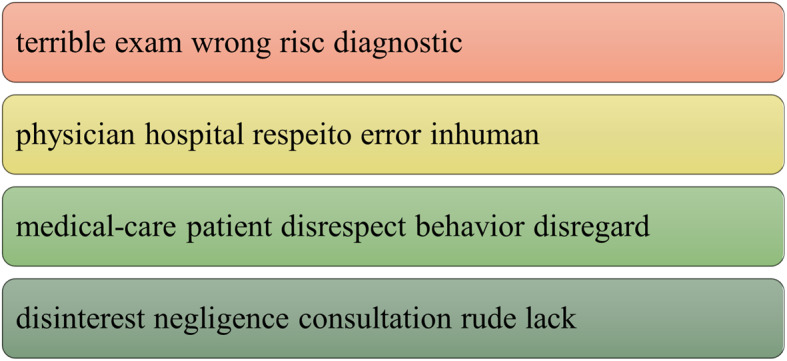
Topics. Source: Voyant Tools [58]

Complaints related to medical care and conduct were more related to emergency care, maternity and hospitalization. Clusters are generated through co-occurrences that group words with greater relation. A cut of 5 words and 4 clusters was made to scale the analysis. (Fig. 2)

In the groupings 4 important fundamentals can be noted. In the first topic there is a relationship between care with wrong and risk. In the second the perception of the physician’s behavior as inhumane. In the third item, the feeling of disrespect and the behavior of the physician and the fourth topic that shows the relationship between the perception of negligence and rude.

The perception of the care, often characterized as “terrible” and inhumane, and the physician’s conduct and behavior, also often characterized as negligent, disrespectful, acting in an arrogant manner, were the reasons that characterized the complaints.

Finding 2: The hostile encounter and the inhospitable encounter.

In assessing the patient’s perception of medical care, Camargo’s conceptual definitions [22, 50] of hostility and inhospitality were considered. Based on these definitions, an analysis of the meanings and keywords of the complaints, the data was grouped into two central categories of the study, representing “not hospitable” care: the hostile encounter and the inhospitable encounter. (Table 1)

**Table 1:** The complaints and evaluation of the meeting

Conceptual definitions	Groupings of complaints	Central categories: evaluation of the meeting
Hostility: the result of aggressive actions that lead to the breakdown of relationships and relational conflicts.	complaints of aggression, violence, harassment, disrespect, arrogance, rudeness,…	Hostile encounter
Inhospitality: an encounter in which the receiver does not recognize and/or ignore the interlocutor; lack of interest in the other.	complaints of negligence, disdain, sloppiness, misconduct, mistake, lack of skill,…	Inhospitable encounter

Source: Created by author

Finding 3: The physician’s behaviors perceived as not hospitable and the patient’s sensations, emotions and feelings.

After evaluating the content of the complaints, we identified two characteristics related to the complaints and related to the physician. Each category was then evaluated concerning its properties, which in this study were defined by the physician’s behaviors as perceived by the patient and the patient’s sensations, emotions, and feelings as reported by the experience.

Most of the complaints were about the physician’s behavior. The behaviors of the physicians observed and reported more frequently were: aggressive, inhumane, disrespectful, arrogant, rude, disinterested, negligent.

In addition, it was possible to identify, from an analytical point of view, the sensations, emotions and feelings that the patients reported as a response to the physician’s behavior during the care. The sensations, emotions and feelings of the patient reported by the experience most often were: disappointment, frustration, anger, dissatisfaction, humiliation, indignation, revolt, fear.

Finding 4: Dimensions of the hostile encounter and the inhospitable encounter.

Using behaviors and sensations as criteria, the reading was once again turned to broadening the characteristics of what was defined as a hostile encounter and an inhospitable encounter. After this evaluation, two dimensions were identified in each of the central categories.

In the hostile encounter a more representative dimension of the physician’s perception of violence and another more representative dimension of the perception of prepotency in relation to the patient. In the inhospitable encounter, the complaints that were more focused on reports of negligence and the other dimension representing the perception of failure in medical conduct were grouped.

Finding 5: Physicians’ attitudes perceived by patients as not hospitable: dehumanization, disregard, dereliction of duty, disability.

With the evaluation of the properties and dimensions of the categories, turned to a careful analysis of the text. With the aim of grouping into nuclei that made sense with the findings to date and, in addition, comparing with the clusters of the textual analysis, groupings organized into codes were made.

The codes represent the physician’s attitudes, which are: Dehumanization, Disregard, Dereliction of duty, and Disability.

Therefore, the physician’s attitudes were identified through a detailed analysis of the complaints that were read and organized, highlighting key words and expressions that represented the physician’s behavior and the sensations, emotions and feelings triggered in the patient and that appeared in the reports about their experiences of medical care. To illustrate each of the attitudes, below are representative excerpts from the patients’ reports:

- Attitude: Dehumanization.

In the office, more violence. I told the physician that I had a pessary, and he turned in the chair to get something, came back to the position in front of me, and said: Yes, I’m going to take it out, he put his hand inside my vagina and pulled it out, without gel, without anesthesia, without a “take a deep breath”. HE JUST TURNED AROUND AND PULLED IT OUT. I was like I was one of those dummies used in college... I wasn’t welcomed, I was physically VIOLATED by the physician’s actions. I WAS deceived, poorly attended to and violated!” (patient 12).

I was attended by a man with an authoritarian and aggressive personality. While I was trying to communicate my problem, he didn’t give me any space to talk, he asked if I wanted to be admitted to a psychiatric department in a threatening tone... He also argued: You’re the patient, I’m the pshysician. A total insult to someone who was already in crisis. I left feeling humiliated (patient 75).

The first few sprays of water into my ears made me feel the malice of this professional if that’s what I can call him! (…) During the procedure I felt a strong discomfort in my eardrum, most likely due to the cold water. So, in addition to the discomfort: the pain; the water running down my body; the rough way in which he injected the water into my ear; the lack of professionalism; and the unwillingness of the PHYSICIAN since the beginning of the service, I couldn’t wait for this service to end because of the entire unpleasant situation. (patient 60)

- Attitude: Disregard.

the physician simply didn’t say a word to me, he communicated with me by sending me away and turning his back, I tried to speak, and he turned his back, continuing with the same hand gesture. I’ve never been treated like this... I had a terrible impression and I’m still feeling sick, I was absurdly embarrassed by everyone looking at me in the room. I felt discriminated (patient 6).

The physician in question called the patients “cutie”, and I could see how uncomfortable the patients seemed with the lack of professionalism... The physician embarrassed me, wouldn’t let me sit down, and attended to me with the door open. (patient 62)

The physician showed he had no medical ethics, respect for the patient, or any desire to provide a minimally acceptable care, it’s like: I’m fed up, I just want to finish my shift... they didn’t take any action so that I wouldn’t fall to the ground and hit my head, as it happened. To top it all off, and attest to his incompetence and total lack of understanding, this citizen who hasn’t learned the minimum to avoid an accident was amused by the situation after the fall. (patient 70)

- Attitude: Dereliction of duty.

Terrible service. Six hours inside an unethical Institution... We were taken from one unit to another where there wasn’t even a physician waiting. It seems compatible with negligence. What if my child has sequelae? (patient 4)

It shows Dereliction of duty, a lack of willingness, a lack of interest in the patient, and a lack of respect for people’s time...It’s not clear whether this is the Physician’s impact or not, but it seems that the Attendant doesn’t want to bother the physician. (patient 16)

- Attitude: Disability.

Wrong pre-diagnosis, didn’t listen to the history reported by the family member, among many others... I recorded a video with the following theme: I’m here at the hospital, I filmed the clock and I said that my son had nothing and that they were going to put him through an unnecessary contrast exam. And his diagnosis was “insecure physician”. (patient 41)

I went to the hospital because of its reputation, but it was the worst choice I ever made: we were victims of a medical error that could have cost my father his life. Thanks to the receptionist at the emergency room who responded to my request for another physician to assess my father, he was not a victim of the incompetence of the physician who initially attended to us in the emergency room reassessment... when we seek medical care, especially when it’s private, the least we need is care and respect for people’s lives! (patient 44)

From the distribution of patients’ complaints about physician’s care, it was possible to assess the frequency with which each attitude appeared in the complaints. In each complaint, it was possible to find more than one attitude related to the patient’s perception. The data shows that the attitude of dereliction of duty was most frequent (39%) in the complaints, followed by disregard (25%), disability (23%) and dehumanization (13%). (Table 2)

**Table 2:** Distribution of complaints according to perception of care and attitudes

Perception of not hospitable care	Attitudes	Number	%
Hostile encounter	Dehumanization	27	13%
	Disregard	53	25%
Inhospitable encounter	Dereliction of duty	83	39%
	Disability	40	23%
		212	100

**Source**: Created by author

Finding 6: Perception of the physician’s attitudes in the hostile encounters: dehumanization, disregard and Perception of the physician’s attitudes in the inhospitable encounter: dereliction of duty, disability.

This finding summarizes the construct of the results, with the groupings, characteristics and definitions of the patient’s perception of medical care in a hospital environment, considered to be not hospitable. (Table 3)

**Table 3:** Summary table of the 4 D’s of non-hospitality

Not hospitable meeting	Cluster	Attitudes: the 4 D’s	Physician’s behavior perceived by patients	The patient’s sensations, emotions and feelings during the meeting
Hostile encounter	C2	Dehumanization	aggression, harassment, violence.	humiliation, mistreatment, violation, threatening condition.
	C3	Disregard	disrespect, arrogance, rudeness, sense of superiority.	indignation, revolt, shame, and embarrassment.
Inhospitable encounter	C4	Dereliction of duty	failing to care for, disregard, lack of interest, and negligence	unwelcome, discomfort, dissatisfaction, frustration.
	C1	Disability	failure in medical conduct, lack of skill or knowledge, avoidance, and insecurity.	fear, anger, disappointment.

Source: Created by author

Following the in-depth analysis of the items of the results.

## Discussion

What is the patient’s perception of medical care, in a hospital environment, that is considered not hospitable?

This article aimed to understand patients’ perceptions of medical care perceived as not hospitable, characterized by a lack of care and welcome, in a hospital environment, based on a complaint’s website.

**Fig. 3:**
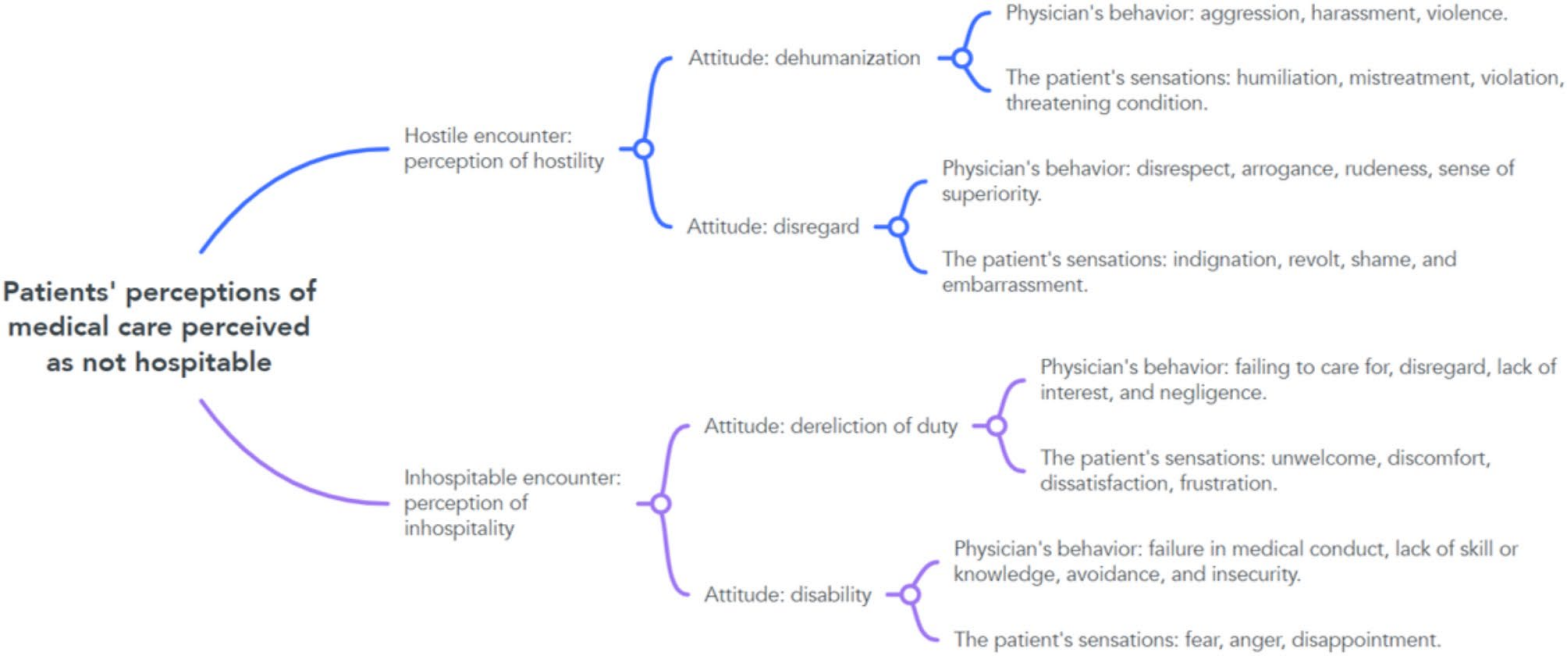
Concept map. Source: Created by author

The encounter perceived by the patient as not hospitable has multiple dimensions, but the attitudes of the physicians were an important criterion of evaluation. In this study the attitudes of the physician were evaluated in relation to the behavior of the physician and the sensations, emotions and feelings, identified by the patients as a response to the meeting. These factors influenced what was characterized as hostile encounter and inhospitable encounter. (Fig. 3)

### The hostile encounter

Hostile encounter have a conflicting nature and have something destructive in the relationship. According to Camargo [22, 50] hostility is the result of aggressive actions that lead to the fraying of human relationships. In any relationship, and particularly in the physician-patient relationship, hostility is harmful and has consequences for the patient and his relationship with his illness process. As the research by Yang and Kirillova [10] and Poorani, Kline, DeMicco and Sullivan [11] points out, it is the physician’s responsibility to create a hospitable space. A meeting perceived as hostile in a hospital that should be a place of welcome and treatment has harmful consequences for the patient’s health. A situation like this could not happen even as a justification considering the precariousness of a health system, as Pazinatto [38] states.

Attitudes perceived by patients as dehumanization and disregard were grouped in this category.

#### Dehumanization

Dehumanization refers to an inhumane attitude characterized by a lack of compassion, empathy, or consideration for others. It is acting in a cruel, insensitive, or disrespectful way.

In hospitality, as Kant [60] points out, the concept of “principle-based benevolence” assumes performing “duties to others” as being attentive, respectful, and careful. For him, this “principle-based benevolence” is a “duty of affability”, a concept that encompasses love and respect for others, united by the law as a duty.

How can there be care and welcome, and even a relationship, when what we have is violence and aggressiveness?

The patients’ reports in this category expressed dehumanization on the part of the physician, as they perceived aggressive, harassing, and violent behavior, triggering feelings of humiliation, violation, and exposure.

The violence presented points out not only the physician’s lack of care but also a perception of “objectification”. To be objectified is to not recognize the other in his humanity and dignity.

There is a lack of recognition of the other, as another individual, in the relationship. For Baptista [61], hospitality, in addition to containing the concept of welcoming, goes further and opens doors to a paradigm of human relationships indexed to the ethical principle of otherness, to the welcoming of the other as ‘Other’.

Not being recognized and not having a place to be valued triggers suffering. Humiliation is a response to harassing behavior that can be perceived as an insult and embarrass a person.

In this type of interaction, in which the brutality of the physician’s perceived behavior reveals cruelty, it is necessary to question what the physician reveals about how the patient should be treated, and who the patient is, to the physician in the interactions.

#### Disregard

The attitude of disregard refers to an attitude of lack of respect, appreciation, or consideration for someone. It is a state of disdain, indifference, or devaluation towards a person.

For Baptista [19] welcoming someone in a hospitable way means opening yourself up without reservations or suspicions. It would be a receptive and trusting attitude and a decisive step toward someone else, making the connection necessary to build a social bond.

Could it be that the attitude of disregard places the other in a place of human devaluation, making it impossible to build bonds?

The patients’ reports in this category expressed disregard for the physician’s attitudes and the behaviors perceived were: disdain, disrespect, arrogance, rudeness, and a sense of superiority. Sensations, emotions, and feelings were: indignation, revolt, shame, and embarrassment.

Shame and embarrassment are feelings provoked by embarrassing situations that show how the physician’s attitudes can reveal the patient’s sense of inadequacy.

If the patient does not feel part of the process and is placed in a position of inferiority by the physician, this breaks down trust and generates suffering.

Disregard in its essence is perverse in the physician-patient interaction because it shows a position that is contrary to the occupation’s ethical principles, which assumes the building of connections, bonds, interactions, and relationships to produce well-being for the patient.

Disregard does not build; it damages and is just as violent as dehumanizing.

### The inhospitable encounter

Unlike the hostile encounter, the inhospitable encounter is, according to Camargo [22] the meeting in which, on the part of the one who receives or the one who is received, the interlocutor is not recognized or is simply ignored. Ignoring the other is not in itself a violent act, but it demonstrates a denial of a relationship, unfriendliness, a desire not to make contact, or, worse, a hidden agenda.

In the physician-patient relationship, the inhospitable encounter supports a troubled and problematic relationship that triggers in the patient feelings of insecurity and not being cared for, which affect the quality of the medical act.

Attitudes perceived by patients as dereliction of duty and disability were grouped in this category.

#### Dereliction of duty

The attitude of dereliction of duty is expressed in a lack of care, attention, or interest in someone. It is a behavior of failing to care for, disregard, lack of interest, or negligence regarding a situation, problem, or need.

Dereliction of duty reinforces the inverse of the welcome, assurance, and reassurance pointed out as the 3-Hs of hospitality by Tasci and Semrad [62], Heart-warming, Heart-assuring, Heart-soothing.

There are no feelings of welcome, protection or reliability.

The patients in this category reported that the physicians were not interested and acted with negligence and disregard. They expressed their sensations, emotions, and feelings about this lack of interest, with discomfort, dissatisfaction, and frustration.

In the patients’ view, physicians fail to provide care that is compatible with the expectation of being cared for and supported.

The dereliction of duty hurts a whole system that should take care and seek quality in terms of health.

#### Disability

The attitude of disability is perceived as a lack of skill, knowledge, or adequate preparation to carry out a certain task or deal with a situation.

From a relational perspective, Gummesson [63] presents four types of interactions that occur in a encounter. The first, and most common, is the interaction between employees and clients, which is the most critical in forming relationships. It depends on the employee’s ability to carry out his activities correctly and properly. For Swartz and Iacobucci [64], the role of employees is very important in high-contact services. Their appearance, behavior, competence, skill, and dedication are essential to perceiving quality.

According to Rossi-Barbosa Lima, Queiroz, Fróes and Caldeira [65], physicians must be attentive to the verbal and non-verbal messages they transmit to patients, especially those transmitted by their posture, gestures, and appearance since these aspects are perceived by the patient and are a determining factor in the feeling of trust.

In the complaints investigated, the frequent behaviors of physicians perceived by patients were: failure in medical conduct, lack of skill or knowledge, avoidance, and insecurity. The sensations, emotions, and feelings triggered were: fear, anger, and disappointment.

Perceived disability, even if not real, is inhospitable because it generates fear and insecurity in the meeting.

Rationalism and the constant attempt at “emotional distance” are still present in the physician’s work. Authors such as Balint [53], Croskerry, Abbass and Wu [66] and Castelhano [66] note that physicians tend to deny or defend themselves against emotional manifestations. This positioning can somehow create a perception of unwelcoming, inhospitality, or even hostility in the patient.

Hospitality is a privileged form of interpersonal encounter and is marked by a welcoming attitude towards others. As it is present in the physician-patient relationship, it should be considered an important criterion for the development and training of physicians and multi-professional teams. Although the focus of the study is the physician, the results can help us understand how attitudes directly influence the patient’s experience. When it comes to the issue of hostility and inhospitability, this is more effective. The findings can direct reflections and actions towards understanding the values of hospitality and understanding its continuum, working bridges to deal with issues of inhospitality. In addition to being an ethical element, hospitality is also interventional.

## Conclusions

The research aimed to understand patients’ perception of the care provided by physicians in the hospital environment, in a meeting perceived as not hospitable, based on a complaint’s registration website.

Based on the analysis of the results, we came up with what we call the 4 D’s of non-hospitality. The most frequent attitude identified was one of dereliction of duty (39%). The 4 D’s can explain and summarize an not hospitable interaction. These attitudes were classified as:

### 1D. Dehumanization

an attitude characterized by a lack of compassion and empathy.

- Physician’s behavior perceived by patients: aggressive, harassing, inhumane, and violent.
- Sensations, emotions, and feelings: humiliation, mistreatment, violation, a threatening condition.

### 2D. Disregard

attitude referring to disdain, depreciation, or lack of respect.

- Physician’s behavior perceived by patients: disrespectful, arrogant, rude, acting superior.
- Sensations, emotions, and feelings: indignation, revolt, shame, embarrassment.

### 3D. Dereliction of duty

an attitude expressed in a lack of care, attention, or interest.

- Physician’s behavior perceived by patients: failing to care for, disregard, lack of interest, and negligence.
- Sensations, emotions, and feelings: unwelcome, discomfort, dissatisfaction, and frustration.

### 4D. Disability

attitude perceived as a lack of preparation and failure in training.

- Physician’s behavior perceived by patients: failure in medical conduct, lack of skill or knowledge, avoidance, and insecurity.
- Sensations, emotions, and feelings: fear, anger, disappointment.

The practical and theoretical implications of this study concern the need to consider hospitality, attitude, and the form of care, which are key factors in the physician’s role in patient treatment and consolidating a health system, especially within hospitals, where the ethics of hospitality must prevail.

The interaction between physician and patient is an important part of the care and health process. When this interaction becomes not hospitable, either by the hostile encounter, or by the inhospitable encounter, the consequences can be harmful to the patient. Physicians have great responsibility in building a system and building a culture of hospitality.

The attitudes of the physician during the consultation can interfere with the patient’s perception and have consequences in the treatment and in the relationship that the patient establishes with his health.

It is urgent to rethink a new model. In their professional work, physicians must dedicate themselves to a practice that considers relational aspects and the responsibilities and implications of interacting with patients. Transformations must be discussed from an educational and institutional point of view, as well as from the point of view of the relationship and perceptions of attitudes so that physicians and patients can build health and well-being strategies together through the bond. This scenario points to the need for further research, especially considering the effects that hostile attitudes could generate and compromise the patient’s health.

The limitations of this study include the fact that we did not have a profile of the sample, with details of the population and the type of hospital care that generated the complaint, since medical care at the hospital can be accessed through multiple services, such as emergency room, consultation, hospitalization, and/or exams.

## Data Availability

The data used in this study was collected on the “Reclame Aqui”, the largest open and independent platform for complaints registration between people and companies in Latin America, available at: https://www.reclameaqui.com.br.
